# Supplementary material for: Adherence and sustainability of interventions informing optimal control against the COVID-19 pandemic
Source: Commun Med (Lond). 2021 Dec 6;1:57. doi: 10.1038/s43856-021-00057-5 (PMC9053235; doi:10.1038/s43856-021-00057-5)
Supplement: Supplementary file 1 — Supplementary Information [file 43856_2021_57_MOESM1_ESM.pdf]

SUPPLEMENTARY INFORMATION

Adherence and sustainability of interventions informing optimal control against the COVID-19 pandemic

Laura Di Domenico[1], Chiara E. Sabbatini[1], Pierre-Yves Boëlle[1], Chiara Poletto[1], Pascal Crépey[2], Juliette Paireau[3,4], Simon Cauchemez[3], François Beck[4], Harold Noel[4], Daniel Lévy-Bruhl[4], Vittoria Colizza[1,5]

[1]*INSERM, Sorbonne Université, Pierre Louis Institute of Epidemiology and Public Health, Paris, France*
[2]*Univ Rennes, EHESP, REPERES « Recherche en Pharmaco-Epidémiologie et Recours aux Soins » – EA 7449, 35043 Rennes, France.*
[3]*Mathematical Modelling of Infectious Diseases Unit, Institut Pasteur, UMR2000, CNRS, Paris, France*
[4]*Santé Publique France, French National Public Health Agency, Saint-Maurice, France*
[5]*Tokyo Tech World Research Hub Initiative, Institute of Innovative Research, Tokyo Institute of Technology, Tokyo, Japan*

Supplementary Methods……….2

Supplementary Note 1: Additional results ……………………………………………………………………………………………3

Supplementary Note 2: Sensitivity analysis……………………………………………………………………………………………4

Supplementary Tables 1-2……….5

Supplementary Figures 1-14…….6

Supplementary References…….16

Supplementary Methods

1. SARS-CoV-2 two-strain transmission model

1.1. Compartmental model and parameters

Supplementary Fig. 1 shows the compartmental scheme used to describe COVID-19 disease progression. Parameter values related to infection due to historical strains are reported in **Supplementary Table 1**. Parameters values for the Alpha variant are presented in the **Methods** section. When vaccination starts, we assumed that doses might be given to either susceptible or recovered individuals with equal probability. Efficacy of vaccination is described in the **Methods** section; such efficacy would only have an impact on the susceptible population.

1.2. Generation time distribution

The generation time distribution was computed based on the approach of Ref.[1]. Let X and Y be the random variables describing the latency period and the infectious period, respectively. Then the distribution of the generation time is the result of the convolution $g * h_s$, with g being the probability density function of X and

$$h_s(t) = \frac{1 - H(t)}{E(Y)}$$

where H is the cumulative distribution function of Y, and $E(Y)$ is the mean.
In the compartmental model under consideration (**Supplementary Fig. S1**), we have that X is exponentially distributed with rate ϵ, and Y is the sum of two exponentially distributed random variables (prodromic phase and infectious period, with rate μ_p and μ respectively). Computations show that the corresponding generation time distribution is

$$f(t) = \frac{\epsilon\, \mu_p\, \mu}{(\mu_p + \mu)(\mu - \mu_p)} \left[\frac{\mu}{(\epsilon - \mu_p)}\left(e^{-\mu_p t} - e^{-\epsilon\, t}\right) - \frac{\mu_p}{(\epsilon - \mu)}\left(e^{-\mu\, t} - e^{-\epsilon\, t}\right)\right]$$

Given the values of ϵ and μ_p informed from the literature (**Supplementary Table 1**), we chose μ so that the mean of the generation time equals to 6.6 days. The shape of the distribution is displayed in **Supplementary Fig. 2** and it closely resembles a gamma distribution with mean 6.6 and shape parameter 1.87, estimated in Ref[2].

1.3. Frequency of the Alpha variant over time

Supplementary Fig. 3 reports the proportion of infections associated with Alpha variant, from genomic (Flash surveys) and virological surveillance data (see **Methods**). Weekly data have been normalized on Alpha and historical strains for comparison with model outcomes, as the two-strains model does not account for additional variants. Model predictions agree well with observed data. Discrepancies between model and data appear in the month of April after strengthened measures were applied, and may be due to the interaction of Alpha (B.1.1.7) with Beta (B.1.351) and Gamma (P.1) variants, slowly expanding their diffusion in the region, and neglected in the model.

The figure also shows the model outcomes obtained assuming that Alpha variant is 40% more transmissible than the historical variants, i.e. the lower estimate provided in Ref[3]. Results show that with this hypothesis the model is not able to capture the evolution in time of frequency of the Alpha variant.

2. Stringency index

The stringency index[4] is a quantity based on multiple indicators that include restrictions on public gatherings, stay-at-home requirements, school closures and travel bans. It provides a measure on the intensity of government policies, with a numerical value ranging from 0 to 100 (a higher value indicates a stricter response). **Supplementary Fig. 4** shows the stringency index for France. If policies vary locally (e.g. at the regional level), the national index corresponds to that of the region with the strictest restrictions. The strengthened measures adopted at the end of March in Île-de-France, are of a similar intensity to the moderate lockdown implemented to curb the second wave.

Supplementary Note 1: Additional results

1. Behavioral indicators vs risk perception and psychosocial burden

Supplementary Fig. 5 reports the correlation analysis between adoption of social distancing and prevalence of anxiety in the population. We observed a non-significant association between these two quantities. The figure also shows that the association found between adoption of social distancing and fear to contract the virus since the second wave (**Fig. 1** of the main paper) holds when extended to the whole time period.

2. Results for different hospitalization levels triggering lockdowns

We show results for different hospitalization levels triggering interventions, i.e. interventions applied at different starting dates. **Supplementary Fig. 6** and **Supplementary Fig. 7** complete the results presented in **Fig. 2** and **Fig. 4** of the main text, referring to interventions applied at week 12, i.e. when weekly hospital admissions reached 2,900.

3. Impact of school holidays

In **Supplementary Fig. 8**, we present the results obtained assuming schools to be open in w15-w16, to evaluate the effect of school holidays in those two weeks. We found that, under moderate interventions, hospitalizations would reach higher peaks and the epidemic would be less easily controlled, if schools were always in session.

4. Impact of different vaccination rhythms

Supplementary Fig. 9 shows the effect of different vaccination rollouts on the scenarios presented in the main text.

5. Phasing out strict lockdowns

Supplementary Fig. 10 shows the impact of a progressive transition in phasing out a 2-week strict lockdown, analogously to results of **Fig. 5** of the main paper for moderate lockdowns. Results support the importance to lower the incidence level to better manage possible rebounds while reopening.

Supplementary Note 2: Sensitivity analysis

Here we present the results of our sensitivity analysis. We test a different value for the transmissibility advantage of the Alpha variant, different assumptions on the delay from the implementation of the restrictions to the peak and different vaccine efficacies.

1. Sensitivity on increase in transmission due to Alpha variant

We assessed the impact of a smaller transmissibility advantage (40% increase vs. 59% increase considered in the main text). Under this assumption, interventions are more effective in controlling the epidemic (**Supplementary Fig. 11**). This assumption is however not compatible with the registered evolution of the Alpha variant in time in the region (see **Supplementary Fig. 3**).

2. Sensitivity on the delay from implementation of the intervention to peak

We assessed the impact of a different delay from the implementation of the intervention to peak, which is set to 7 days in the main text, based on estimates from lockdowns implemented in 2020. Results remained robust: assuming a 10-day delay leads to differences in the ballpark of estimations (**Supplementary Fig. 12**).

3. Vaccine efficacy

Supplementary Fig. 13 reports the sensitivity on vaccine efficacy against transmission. We tested 40% vaccine efficacy against transmission, with respect to 65% efficacy assumed in the main text. We found no significant differences in hospital admission trajectories, owing to an already high efficacy against contracting the infection, playing a major role in reducing epidemic activity. **Supplementary Fig. 14** shows the results of the sensitivity on the delay between vaccine administration and vaccine efficacy (2 weeks vs. 3 weeks considered in the main results). As expected, a shorter delay anticipates the effect of vaccination on the epidemic evolution, but does not affect the general results presented in the main text.

Supplementary Table 1. Parameters, values, and sources used to define the compartmental model for infection due to historical strains in absence of vaccines.

**Variable**	**Description**	**Value**	**Source**
θ^{-1}	Incubation period	5.2d	[5]
μ_p^{-1}	Duration of prodromal phase	1.5d, computed as the fraction of pre-symptomatic transmission events out of pre-symptomatic plus symptomatic transmission events.	[6]
ϵ^{-1}	Latency period	$\theta^{-1} - \mu_p^{-1}$	-
p_a	Probability of being asymptomatic	0.4	[7]
p_{ps}	If symptomatic, probability of being paucisymptomatic	1 for children, adolescents<br>0.2 for adults, seniors	[8]
p_{ms}	If symptomatic, probability of developing mild symptoms	0 for children, adolescents<br>0.7704 for adults<br>0.546 for seniors	[8–10]
p_{ss}	If symptomatic, probability of developing severe symptoms	0 for children, adolescents<br>0.0296 for adults<br>0.254 for seniors	[9,10]
g	Generation time	6.6d	[2]
μ^{-1}	Infectious period	2.3d, chosen accordingly to generation time distribution	-
r_β	Relative infectiousness of I_p, I_a, I_{ps}	0.25 for children<br>0.55 for adolescents, adults, seniors	Assumed, in line with available evidence[11–13]
s	Relative susceptibility	0.5 for children, adolescents<br>1 for adults, seniors	[14,15]

Supplementary Table 2. **Distress index associated to lockdown scenarios with different duration, intensity and adherence.** Distress index corresponding to curfew conditions lasting 2 to 8 weeks is shown for comparison.

Intervention	Distress index			
	2 weeks	4 weeks	6 weeks	8 weeks
Strict LD	2.50	5.00	7.50	10.00
Strict LD w/ limited loss of adherence	-	4.51	6.53	8.54
Strict LD w/ continuous loss of adherence	-	-	6.14	7.45
Moderate LD	1.55	3.09	4.64	6.19
Moderate LD w/ limited loss of adherence	-	2.79	4.04	5.29
Moderate LD w/ continuous loss of adherence	-	-	3.80	4.61
Curfew	0.92	1.84	2.76	3.67

Supplementary Figure 1. Two-strains compartmental scheme with vaccination. Compartments with continuous line (top) account for infections due to historical strains, compartments with dashed line (bottom) account for infections due to the Alpha variant. Analogous compartments are considered for vaccinated individuals (not shown for the sake of visualization). S=Susceptible, E=Exposed, I_p= Infectious in the prodromic phase, I_{as}=Asymptomatic Infectious, I_{ps}=Paucysymptomatic Infectious, I_{ms}=Symptomatic Infectious with mild symptoms, I_{ss}=Symptomatic Infectious with severe symptoms, H=severe case admitted to the hospital, R=Recovered.

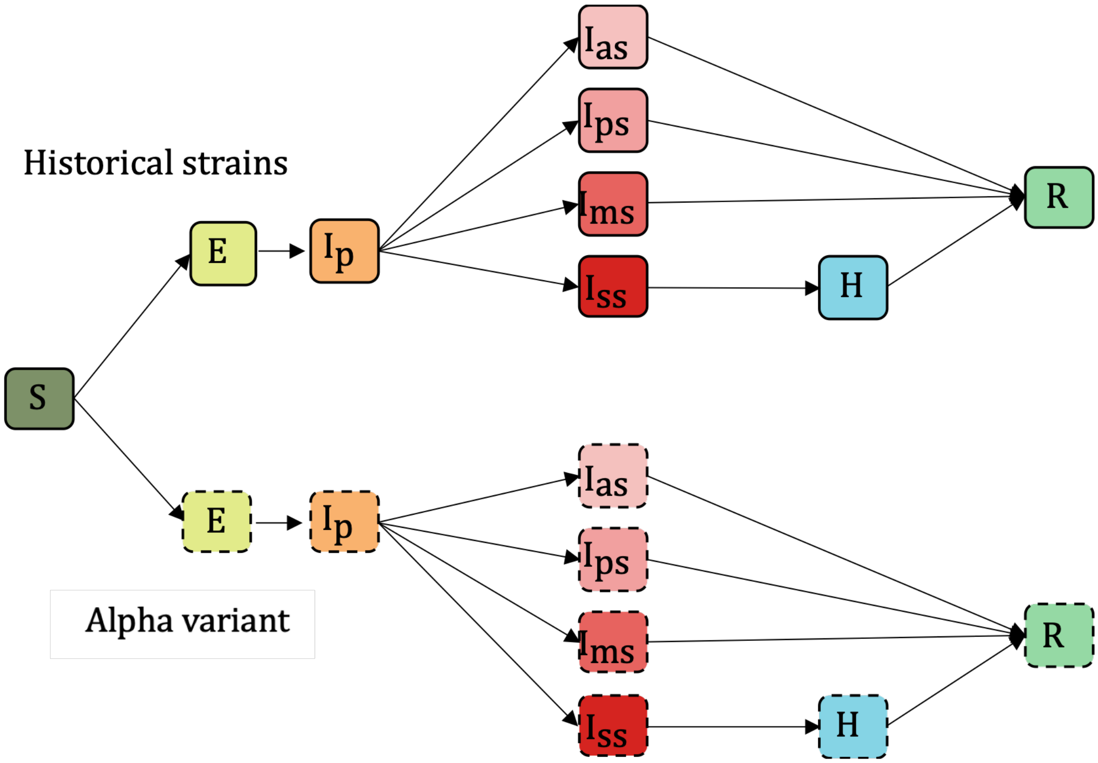

Supplementary Figure 2. Distribution of the generation time. The generation time distribution corresponding to our compartmental model (blue) in comparison with the distribution estimated in Ref.[2] (orange).

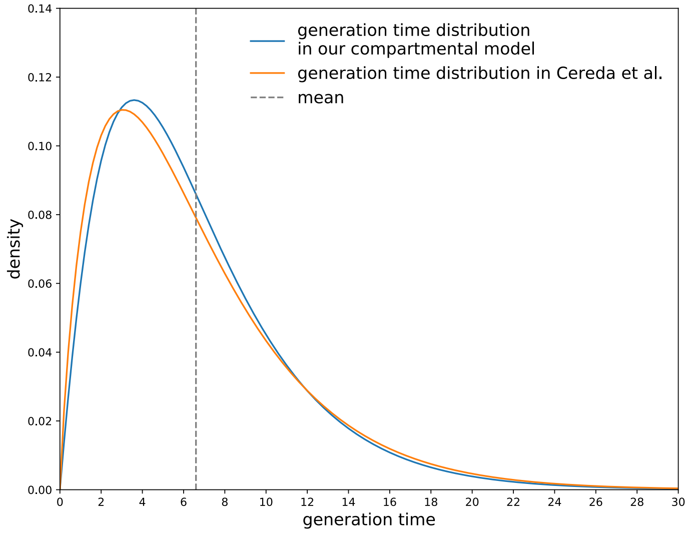

Supplementary Figure 3. Prevalence of Alpha variant over time. Estimated percentage of Alpha cases in Île-de-France over time, considering a 59% (95% CI: 54–65%) higher transmissibility (red) and a 40% higher transmissibility (blue) for the variant. Circles represent the estimates from the genomic surveillance in the Flash surveys (Flash1 on 7–8 January, Flash2 on 27 January, Flash3 on 16 February). Squares represent results from weekly virological surveillance screening allowing the detection of the N501Y mutation specific to the Alpha variant. We estimated 95% CI assuming a normal distribution. Flash3 survey estimates have larger CI as sequencing was performed on a smaller sample of viruses. Horizontal bars in weekly virological surveillance correspond to the week of reference.

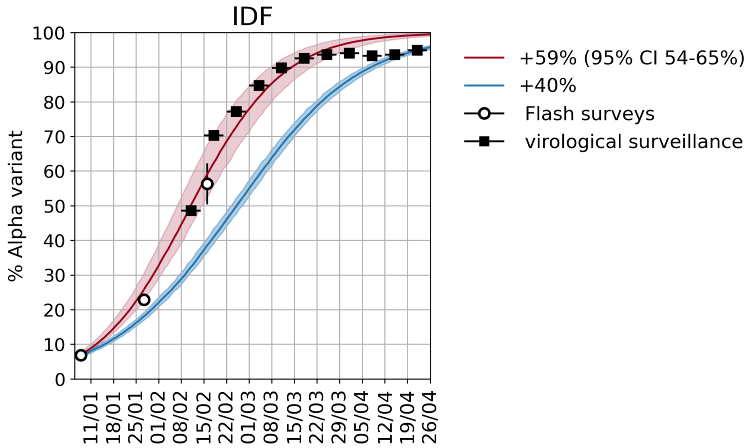

Supplementary Figure 4. Stringency index in France. Estimated stringency index for France over time. Shaded rectangles in the plot correspond to social distancing measures applied during the three waves (strict lockdown in the first wave, moderate lockdown in the second wave, strengthened measures in the third wave).

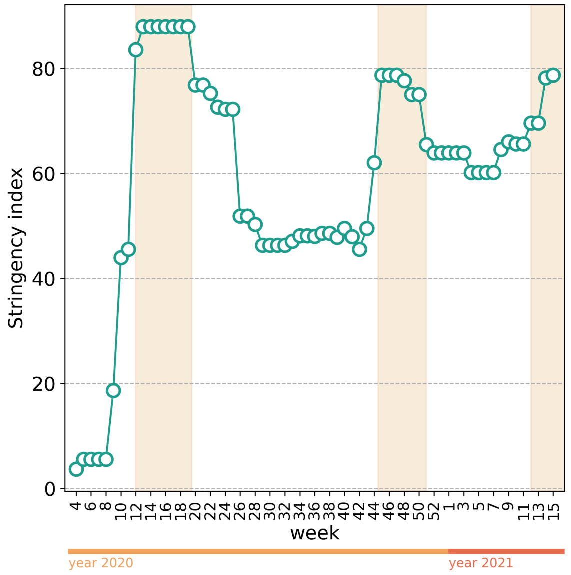

Supplementary Figure 5. Risk perception, social distancing, anxiety during COVID-19 pandemic. Prevalence of anxiety in the context of COVID-19 pandemic (left)[16] as functions of time; scattered plot between the prevalence of anxiety and the percentage of individuals avoiding crowded public places[17] in the time period March 2020 - April 2021, with the results of a Pearson correlation test, effect size 0.2, p-value 0.46 (center). Scattered plot between the fear to contract COVID-19 and the percentage of individuals avoiding crowded public in the time period March 2020 - April 2021, with the results of a Pearson correlation test, effect size 0.88, p-value < 10^{-3} (right). Results for these indicators refer to the national scale.

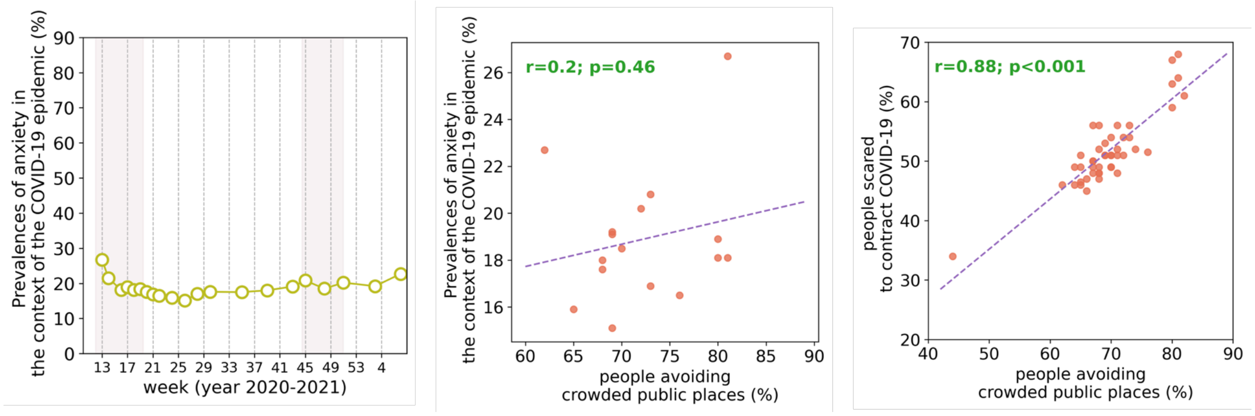

Supplementary Figure 6. Impact of timing of interventions on the timecourse of weekly hospital admissions. From left to right: increasing levels of weekly hospital admissions triggering lockdowns, corresponding to implementing interventions starting from w11 up to w15. From top to bottom: increasing lockdown duration (weeks). Solid curves refer to the median overall trajectory, obtained under the vaccination rollout of 300k first doses administered per day starting April. Dashed curves show the same for an accelerated vaccination rhythm (500k first doses/day starting April). The shaded area around the curves corresponds to the 95% probability range obtained from n=250 stochastic simulations. The type of intervention is coded by different line colors. Dots refer to data; filled dots correspond to the data used to fit the model and to provide the trajectory for the curfew scenario; void dots correspond to more recent data. Horizontal dashed lines refer to the peak of the first and second wave in the region. A 7-day delay is assumed from the implementation of the intervention to the peak.

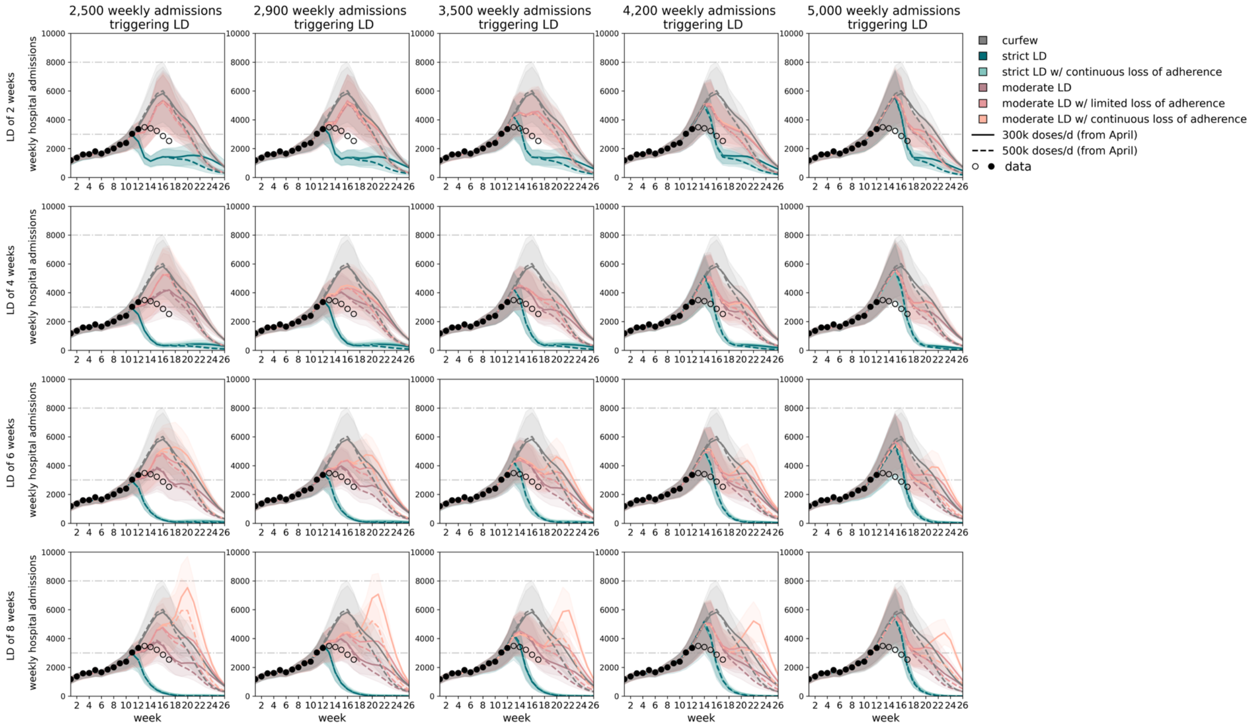

Supplementary Figure 7. Impact of timing of interventions on the intervention efficacy and associated policy-induced distress. From left to right: weekly hospital admissions at the end of June (w26), cumulative hospital admissions (computed in the time period w12-w26), hospital pressure, defined as the number of weeks in which hospital admissions remain above the peak level achieved during the second wave, in the period w12-w26 as functions of the distress index. From top to bottom: increasing levels of weekly hospital admissions triggering lockdowns corresponding to implementing interventions in w11, w13, w14 or w15. Results refer to the accelerated vaccination pace of 300,000 first doses/day since April. Color shades of the symbol contour refer to the duration (weeks) of the lockdown intervention (from the lightest shade corresponding to 2 weeks, to the darkest one corresponding to 8 weeks). Adherence to moderate and strict lockdowns is coded with the fill color (filled symbols with the color of the scenario correspond to scenarios with full adherence, void symbols represent scenarios with limited loss in adherence, blue filled-in symbols correspond to scenarios with continuous loss in adherence).

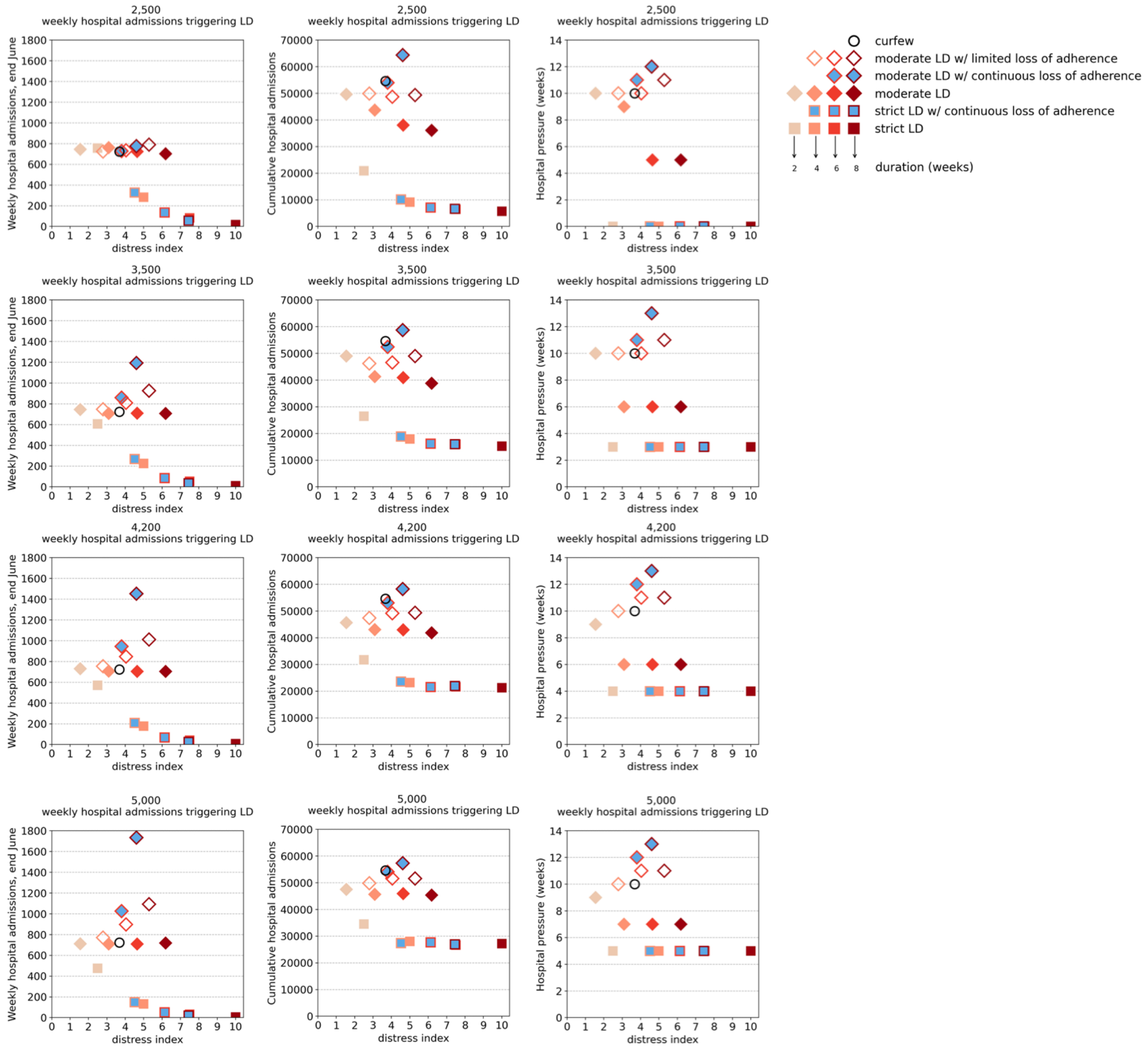

Supplementary Figure 8. Impact of school holidays on the timecourse of weekly hospital admissions in Île-de-France for interventions of varying intensity, duration, and adherence. From left to right: increasing lockdown duration, expressed in weeks. Top row: vaccination pace accelerated to 300,000 first doses/day since the start of April; bottom row: 500,000 first doses/day. Interventions are applied in w12 and assume a delay of one week to the peak in hospital admissions. Schools are assumed to be open in w15-16. Dots refer to data; filled dots correspond to the data used to fit the model and to provide the trajectory for the curfew scenario; void dots correspond to more recent data. Curves refer to the median trajectory; shaded areas around the curves correspond to the 95% probability range obtained from n=250 stochastic simulations. The type of intervention is coded by different line colors. Horizontal dashed lines refer to the peak of the first and second wave in the region. Results for strict lockdown scenarios with full adherence or loss of adherence overlap. For this reason, we do not show the scenario with limited loss of adherence.

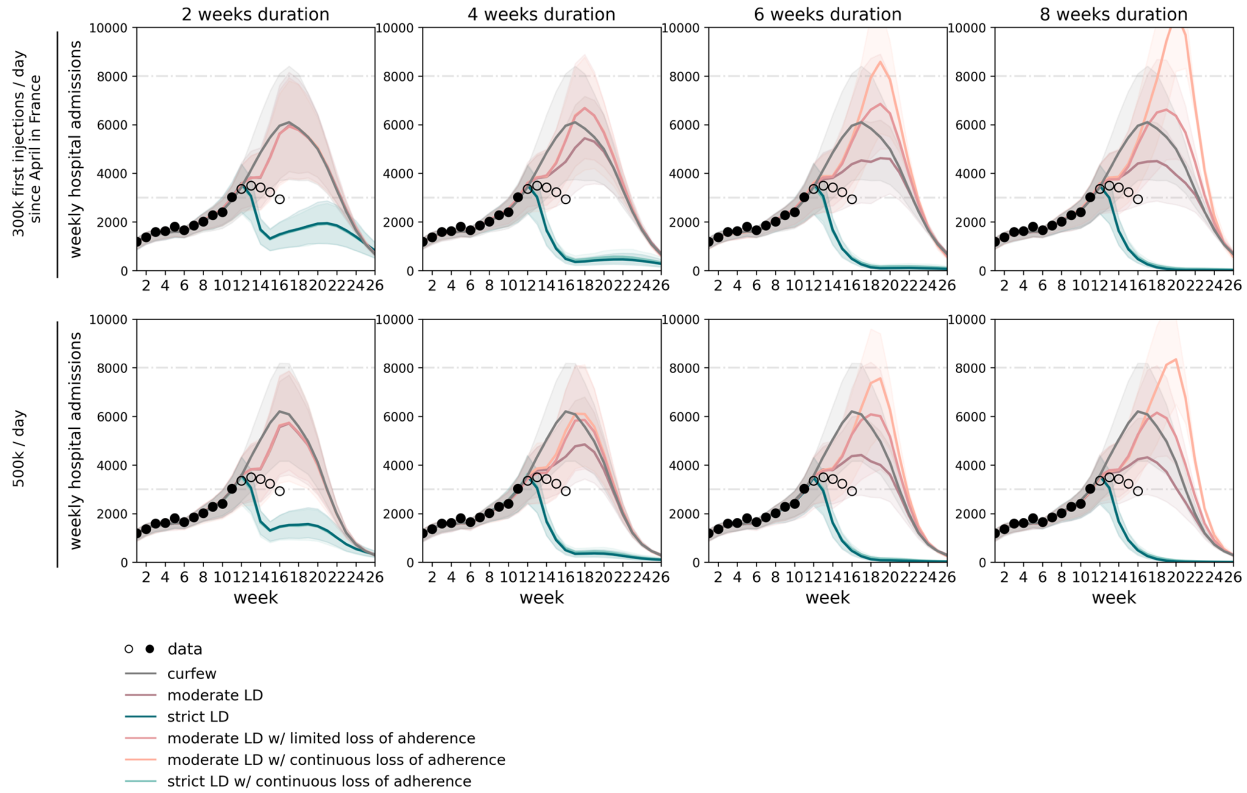

Supplementary Figure 9. Impact of accelerated vaccination. Results refer to interventions applied in w12. From left to right: weekly hospital admissions at the end on June (w26), cumulative hospital admissions (computed in the time period w12-w26). All outcomes are shown as functions of the vaccination rhythm (doses/day since April). Color shades of the symbol contour refer to the duration (weeks) of the lockdown intervention (from the lightest shade corresponding to 4 weeks, to the darkest one corresponding to 8 weeks). Adherence to moderate and strict lockdowns is coded with the fill color (filled symbols with the color of the scenario correspond to scenarios with full adherence, void symbols represent scenarios with limited loss in adherence, blue filled-in symbols correspond to scenarios with continuous loss in adherence). Plots show median values; error bars represent 95% probability obtained from $n = 250$ independent stochastic runs.

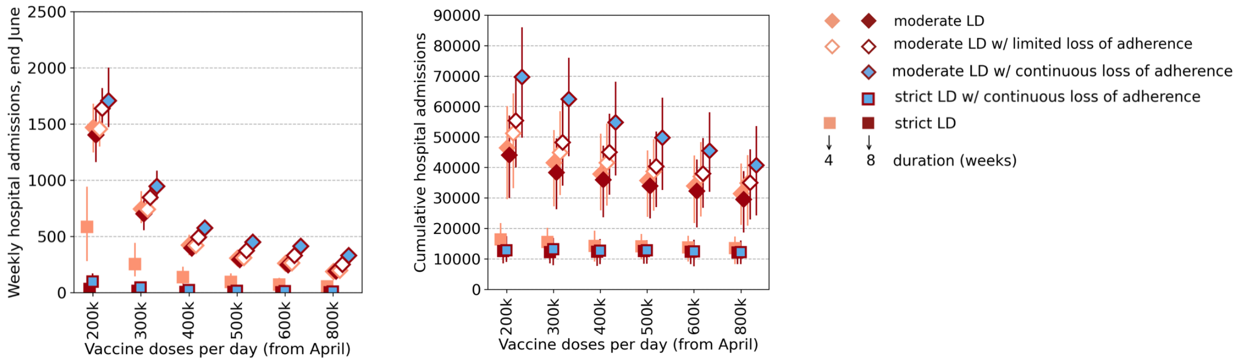

Supplementary Figure 10. Impact of different exit conditions after a 2-week strict lockdown on the timecourse of weekly hospital admission. Plots show projections of the weekly hospital admissions under different hypotheses for the reopening conditions. Progressive transition after lockdown is modeled with 4 weeks of curfew (lighter line) or 6 weeks of curfew (darker line) followed by exit conditions experienced in mid-July 2020, but with schools in session (left); curfew scenarios with 40% fewer individuals respecting physical distancing (center); curfew scenarios with 15% fewer individuals respecting physical distancing (right). Scenarios assume a 10% reduction in transmissibility due to seasonality (except for the mid-July 2020 conditions that already embed seasonal aspects) and a vaccination rhythm of 300,000 first doses per day starting April. Curves refer to the median overall trajectory and the shaded area around the curves corresponds to the 95% probability range obtained from n=250 stochastic simulations.

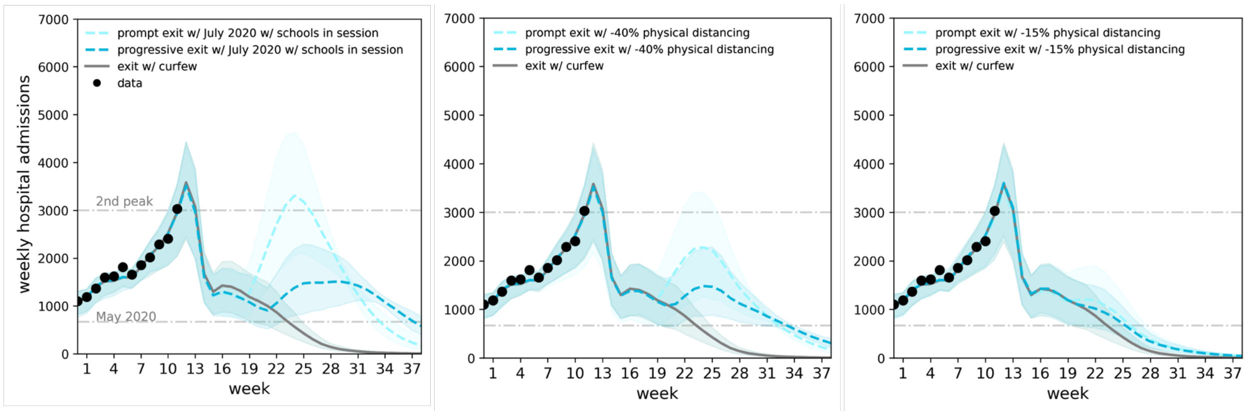

Supplementary Figure 11. Projected impact of interventions on the healthcare system, assuming a 40% transmission increase for Alpha strain. Results refer to interventions applied in w12. From left to right: increasing lockdown duration (weeks). Curves refer to the median overall trajectory, obtained under the vaccination pace of 300k first doses administered per day starting April. The shaded area around the curves corresponds to the 95% probability range obtained from n=250 stochastic simulations. The type of intervention is coded by different line colors. Line type indicates increase in transmission due to Alpha infection; 59% solid line vs 40% dotted line. Dots refer to data; filled dots correspond to the data used to fit the model and to provide the trajectory for the curfew scenario; void dots correspond to more recent data. Horizontal dashed line refers to the peak of the second wave in the region. A 7-day delay is assumed from the implementation of the intervention to the peak.

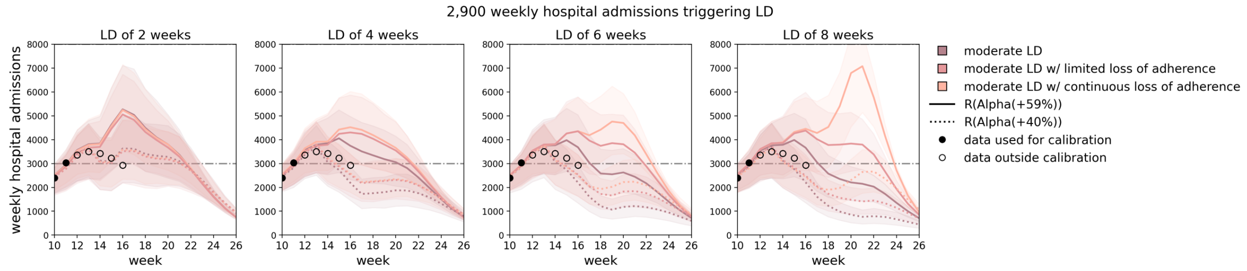

Supplementary Figure 12. **Impact of a longer delay in peak after restriction implementation on the timecourse of weekly hospital admissions.** From left to right: increasing levels of weekly hospital admissions triggering lockdowns corresponding to implementing interventions in w11 to w15. From top to bottom: increasing lockdown duration (weeks). Curves refer to the median overall trajectory, obtained under the vaccination pace of 300k first doses administered per day starting April. The shaded area around the curves corresponds to the 95% probability range obtained from n=250 stochastic simulations. The type of intervention is coded by different line colors. Line type indicates the assumed delay from the implementation of the intervention to the peak; solid line corresponds to a 7 day-delay, dotted-dashed line corresponds to a 10-day delay. Dots refer to data; filled dots correspond to the data used to fit the model and to provide the trajectory for the curfew scenario; void dots correspond to more recent data. Horizontal dashed line refers to the peak of the second wave in the region.

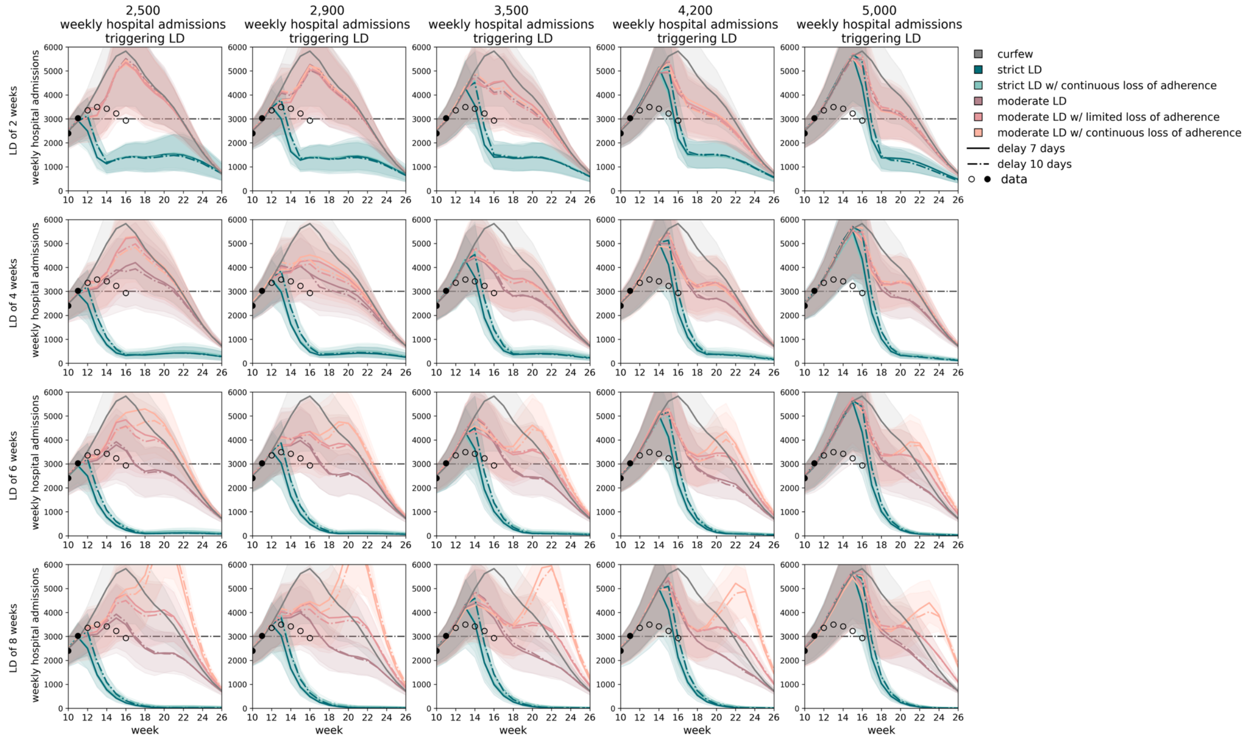

Supplementary Figure 13. **Impact of vaccine efficacy against transmission on the timecourse of weekly hospital admissions** Results refer to interventions applied in w12. From left to right: increasing lockdown duration (weeks). Curves refer to the median overall trajectory, obtained under the vaccination pace of 300k first doses administered per day starting April. The shaded area around the curves corresponds to the 95% probability range obtained from n=250 stochastic simulations. The type of intervention is coded by different line colors. Line type indicates different vaccine efficacies; dashed line is obtained assuming 75% vaccine efficacy against susceptibility, 40% vaccine efficacy against transmission and 80% vaccine efficacy against symptoms given infection (75%-40%-80%); solid line is obtained assuming 75% efficacy against susceptibility, 65% vaccine efficacy against transmission, and 80% vaccine efficacy against symptoms given infection (75%-65%-80%). Dots refer to data; filled dots correspond to the data used to fit the model and to provide the trajectory for the curfew scenario; void dots correspond to more recent data. Horizontal dashed line refers to the peak of the second wave in the region. A 7-day delay is assumed from the implementation of the intervention to the peak.

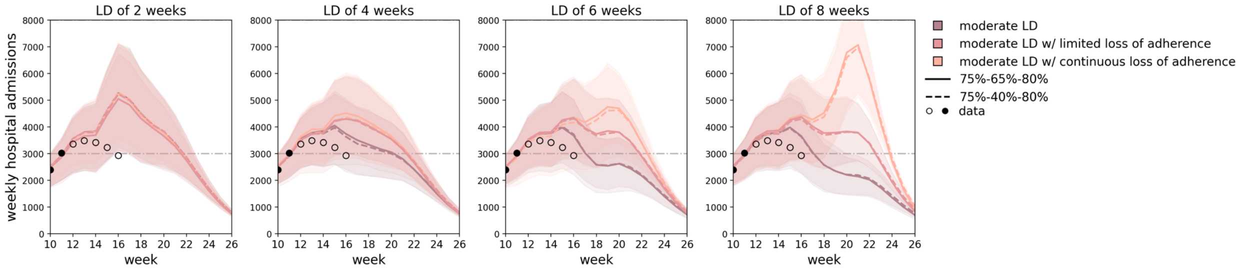

Supplementary Figure 14. **Impact of delay in vaccine efficacy on the timecourse of weekly hospital admissions** Results refer to interventions applied in w12. From left to right: increasing lockdown duration (weeks). Curves refer to the median overall trajectory, obtained under vaccination pace of 300k first doses administered per day starting April. The shaded area around the curves corresponds to the 95% probability range obtained from n=250 stochastic simulations. The type of intervention is coded by different line colors. Curves are obtained assuming a 2-week delay in vaccine efficacy. Dots refer to data; filled dots correspond to the data used to fit the model and to provide the trajectory for the curfew scenario; void dots correspond to more recent data. Horizontal dashed line refers to the peak of the second wave in the region. A 7-day delay is assumed from the implementation of the intervention to the peak.

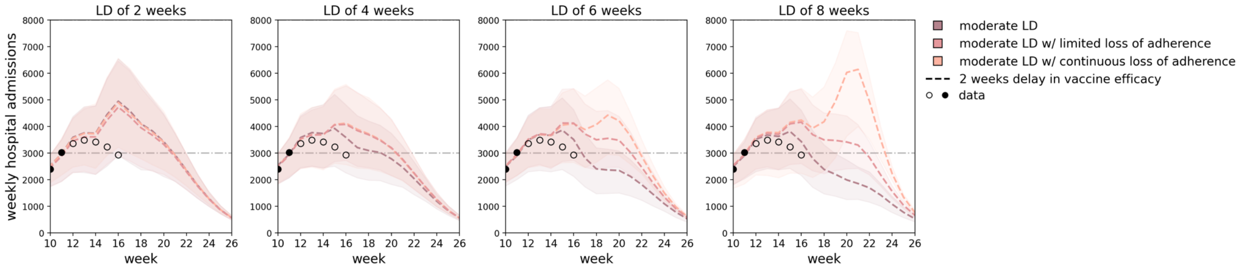

Supplementary References

1. Svensson, A. A note on generation times in epidemic models. *Math. Biosci.* **208**, 300–311 (2007).
2. Cereda, D. *et al.* The early phase of the COVID-19 outbreak in Lombardy, Italy. *arXiv:2003.09320 [q-bio]* http://arxiv.org/abs/2003.09320 (2020).
3. Davies, N. G. *et al.* Estimated transmissibility and impact of SARS-CoV-2 lineage B.1.1.7 in England. *Science* **372**, (2021).
4. Hale, T. *et al.* A global panel database of pandemic policies (Oxford COVID-19 Government Response Tracker). *Nat. Hum. Behav.* **5**, 529–538 (2021).
5. Lauer, S. A. *et al.* The Incubation Period of Coronavirus Disease 2019 (COVID-19) From Publicly Reported Confirmed Cases: Estimation and Application. *Ann. Intern. Med.* **172**, 577–582 (2020).
6. Ferretti, L. *et al.* Quantifying SARS-CoV-2 transmission suggests epidemic control with digital contact tracing. *Science* **368**, eabb6936 (2020).
7. Lavezzo, E. *et al.* Suppression of a SARS-CoV-2 outbreak in the Italian municipality of Vo'. *Nature* **584**, 425–429 (2020).
8. Riccardo, F. *et al.* Epidemiological characteristics of COVID-19 cases and estimates of the reproductive numbers 1 month into the epidemic, Italy, 28 January to 31 March 2020. *Eurosurveillance* **25**, 2000790 (2020).
9. Salje, H. *et al.* Estimating the burden of SARS-CoV-2 in France. *Science* **369**, 208–211 (2020).
10. Lapidus, N. *et al.* Do not neglect SARS-CoV-2 hospitalization and fatality risks in the middle-aged adult population. *Infect. Dis. Now* (2021) doi:10.1016/j.idnow.2020.12.007.
11. Goldstein, E., Lipsitch, M. & Cevik, M. On the Effect of Age on the Transmission of SARS-CoV-2 in Households, Schools, and the Community. *J. Infect. Dis.* **223**, 362–369 (2021).
12. Galmiche, S. *et al.* Etude des facteurs sociodémographiques, comportements et pratiques associés à l'infection par le SARS-CoV-2 (ComCor). https://hal-pasteur.archives-ouvertes.fr/pasteur-03155847 (2021).
13. Li, R. *et al.* Substantial undocumented infection facilitates the rapid dissemination of novel coronavirus (SARS-CoV2). *Science* (2020) doi:10.1126/science.abb3221.
14. Davies, N. G. *et al.* Age-dependent effects in the transmission and control of COVID-19 epidemics. *Nat. Med.* **26**, 1205–1211 (2020).
15. Viner, R. M. *et al.* Susceptibility to SARS-CoV-2 Infection Among Children and Adolescents Compared With Adults: A Systematic Review and Meta-analysis. *JAMA Pediatr.* **175**, 143 (2021).
16. Santé Publique France. CoviPrev : une enquête pour suivre l'évolution des comportements et de la santé mentale pendant l'épidémie de COVID-19. https://www.santepubliquefrance.fr/etudes-et-enquetes/coviprev-une-enquete-pour-suivre-l-evolution-des-comportements-et-de-la-sante-mentale-pendant-l-epidemie-de-covid-19 (2020).
17. YouGov.co.uk. COVID-19 Public Monitor. https://yougov.co.uk/covid-19 (2021).
