# Supplementary material for: Adherence and sustainability of interventions informing optimal control against the COVID-19 pandemic
Source: Commun Med (Lond). 2021 Dec 6;1:57. doi: 10.1038/s43856-021-00057-5 (PMC9053235; doi:10.1038/s43856-021-00057-5)
Supplement: Supplementary file 2 — Description of Additional Supplementary Files [file 43856_2021_57_MOESM2_ESM.pdf]

Description of Additional Supplementary Files

File Name: Supplementary Data 1

Description: Source data for Figure 1

File Name: Supplementary Data 2

Description: Source data for Figure 2

File Name: Supplementary Data 3

Description: Source data for Figure 3

File Name: Supplementary Data 4

Description: Source data for Figure 4

File Name: Supplementary Data 5

Description: Source data for Figure 5
