# Supplementary material for: Adherence and sustainability of interventions informing optimal control against the COVID-19 pandemic
Source: Commun Med (Lond). 2021 Dec 6;1:57. doi: 10.1038/s43856-021-00057-5 (PMC9053235; doi:10.1038/s43856-021-00057-5)
Supplement: Supplementary file 9 — Reporting Summary [file 43856_2021_57_MOESM9_ESM.pdf]

nature research

Corresponding author(s): Vittoria Colizza

Last updated by author(s): 2021/11/02

Reporting Summary

Nature Research wishes to improve the reproducibility of the work that we publish. This form provides structure for consistency and transparency in reporting. For further information on Nature Research policies, see our Editorial Policies and the Editorial Policy Checklist.

Statistics

For all statistical analyses, confirm that the following items are present in the figure legend, table legend, main text, or Methods section.

n/a	Confirmed	
☐	☒	The exact sample size (n) for each experimental group/condition, given as a discrete number and unit of measurement
☒	☐	A statement on whether measurements were taken from distinct samples or whether the same sample was measured repeatedly
☐	☒	The statistical test(s) used AND whether they are one- or two-sided<br>*Only common tests should be described solely by name; describe more complex techniques in the Methods section.*
☒	☐	A description of all covariates tested
☐	☒	A description of any assumptions or corrections, such as tests of normality and adjustment for multiple comparisons
☐	☒	A full description of the statistical parameters including central tendency (e.g. means) or other basic estimates (e.g. regression coefficient) AND variation (e.g. standard deviation) or associated estimates of uncertainty (e.g. confidence intervals)
☐	☒	For null hypothesis testing, the test statistic (e.g. F, t, r) with confidence intervals, effect sizes, degrees of freedom and P value noted<br>*Give P values as exact values whenever suitable.*
☒	☐	For Bayesian analysis, information on the choice of priors and Markov chain Monte Carlo settings
☒	☐	For hierarchical and complex designs, identification of the appropriate level for tests and full reporting of outcomes
☐	☒	Estimates of effect sizes (e.g. Cohen's d, Pearson's r), indicating how they were calculated

Our web collection on statistics for biologists contains articles on many of the points above.

Software and code

Policy information about availability of computer code

Data collection	Computer code was not used to collect data.
Data analysis	Analyses were carried out in Python 3.8.5. Code for the transmission model is available at https://doi.org/10.5281/zenodo.5603812

For manuscripts utilizing custom algorithms or software that are central to the research but not yet described in published literature, software must be made available to editors and reviewers. We strongly encourage code deposition in a community repository (e.g. GitHub). See the Nature Research guidelines for submitting code & software for further information.

Data

Policy information about availability of data

All manuscripts must include a data availability statement. This statement should provide the following information, where applicable:

- Accession codes, unique identifiers, or web links for publicly available datasets
- A list of figures that have associated raw data
- A description of any restrictions on data availability

The mobility data supporting the findings of this study were available to authors from the Orange Business Service Flux Vision within the framework of the research project ANR EVALCOVID-19 (ANR-20-COVI-0007). Restrictions apply to the availability of these data, which were used under license, and so are not publicly available. Access to the data can be requested from Orange Business Service Flux Vision on a contractual basis. All other indicators used in the study are publicly available online at the links provided in the references (see reference list in the main article). Hospitalization data were obtained from the SIVIC dataset[7]. Presence at workplaces was obtained from Google Mobility Reports[15] specific to Île-de-France region. Indicators of social distancing ("Avoiding crowded public places")[69] and risk perception ("% people who say they are 'very' or 'somewhat' scared that they will contract COVID-19")[70] were obtained from YouGov.uk. Data on mental health were obtained from Santé publique France[17], in the section "Santé mental - Prévalences et évolutions de l'anxiété". Source data for the main figures in the manuscript can be accessed as Supplementary Data 1-5.

Field-specific reporting

Please select the one below that is the best fit for your research. If you are not sure, read the appropriate sections before making your selection.

☒ Life sciences ☐ Behavioural & social sciences ☐ Ecological, evolutionary & environmental sciences

For a reference copy of the document with all sections, see nature.com/documents/nr-reporting-summary-flat.pdf

Life sciences study design

All studies must disclose on these points even when the disclosure is negative.

Sample size	We averaged 250 stochastic simulations to compute the average expected value for the likelihood and for all outcomes of interest.
Data exclusions	The study focuses on the region of Ile-de-France, as one of the most affected regions hitted by COVID-19 epidemic and the region with the highest penetration of B.1.1.7 at the start of 2020. Other French regions were excluded from the study.
Replication	Each model replication of the stochastic epidemic model was independently executed, by assigning a distinct seed to initialize the random number generator.
Randomization	Our study is observational and population-based in nature, and involves no intervention. Randomization of subjects is therefore not applicable.
Blinding	Our study is observational and population-based in nature, and involves no intervention. Blinding techniques are therefore not applicable.

Reporting for specific materials, systems and methods

We require information from authors about some types of materials, experimental systems and methods used in many studies. Here, indicate whether each material, system or method listed is relevant to your study. If you are not sure if a list item applies to your research, read the appropriate section before selecting a response.

Materials & experimental systems

n/a	Involved in the study	
☒	☐	Antibodies
☒	☐	Eukaryotic cell lines
☒	☐	Palaeontology and archaeology
☒	☐	Animals and other organisms
☒	☐	Human research participants
☒	☐	Clinical data
☒	☐	Dual use research of concern

Methods

n/a	Involved in the study	
☒	☐	ChIP-seq
☒	☐	Flow cytometry
☒	☐	MRI-based neuroimaging
